# Polygenic Risk Score as a Predictor of Bone Fracture or Osteoporosis in Prostate Cancer Patients Receiving Androgen Deprivation Therapy

**DOI:** 10.1002/cam4.71395

**Published:** 2025-11-20

**Authors:** Ying‐Qiang Liu, Li‐Wen Chang, Hui‐Wen Yang, Jian‐Ri Li, Yi‐Ming Chen, Sheng‐Chun Hung, I‐Chieh Chen

**Affiliations:** ^1^ Department of Medical Education Taichung Veterans General Hospital Taichung Taiwan; ^2^ Department of Physical Medicine and Rehabilitation Taichung Veterans General Hospital Taichung Taiwan; ^3^ Department of Urology Taichung Veterans General Hospital Taichung Taiwan; ^4^ Department of Post‐Baccalaureate Medicine, College of Medicine National Chung Hsing University Taichung Taiwan; ^5^ Institute of Medicine Chung Shan Medical University Taichung Taiwan; ^6^ Department of Medical Research Taichung Veterans General Hospital Taichung Taiwan; ^7^ Department of Medicine and Nursing Hungkuang University Taichung Taiwan; ^8^ Division of Allergy, Immunology and Rheumatology Taichung Veterans General Hospital Taichung Taiwan; ^9^ Graduate Institute of Clinical Medicine, College of Medicine National Chung Hsing University Taichung Taiwan; ^10^ Precision Medicine Research Center, College of Medicine National Chung Hsing University Taichung Taiwan; ^11^ School of Medicine National Yang‐Ming Chiao Tung University Taipei Taiwan; ^12^ Master Program in Precision Health National Chung Hsing University Taichung Taiwan

**Keywords:** androgen deprivation therapy, bone fracture, incidence, osteoporosis, polygenic risk score, prostate cancer

## Abstract

**Background:**

The association between single nucleotide polymorphisms (SNPs) and fracture risk has been established in various studies. Androgen‐deprivation therapy (ADT) for prostate cancer, while extending survival, increases the risk of osteoporosis and bone fracture. This study aimed to assess the predictive value of polygenic risk scores (PRSs) on bone events among prostate cancer patients undergoing ADT.

**Methods:**

We enrolled 24,778 male participants, including 903 prostate cancer patients treated at Taichung Veterans General Hospital. These patients were divided into ADT and non‐ADT groups. Nine PRSs related to bone mineral density from the PGS Catalog were tested and categorized into quartiles. Cox proportional hazards regression analysis was performed to evaluate the risk of bone fracture and osteoporosis between groups by PRS quartiles.

**Results:**

Among the PRSs, PGS002632, PGS002681, and PGS001955 demonstrated the highest areas under the curve (AUCs) for predicting bone events. For PGS002632, the risk was significantly higher in the first quartile (Q1) compared to the fourth quartile (Q4) for patients receiving ADT (HR = 5.934, *p* = 0.0013), after adjusting for age and comorbidities. Furthermore, within the subgroup of prostate cancer patients in Q1 of PGS002632, the risk was higher for those receiving ADT compared to those not receiving ADT (HR = 3.698, *p* = 0.0093). Similar results were observed for PGS002681 and PGS001955.

**Conclusion:**

This hospital‐based cohort study among Han Chinese highlights PRS as a potential biomarker for bone fracture and osteoporosis risk in prostate cancer patients initiating ADT. It provides a basis for personalized risk assessment and early intervention with bone protective strategies.

## 1 Introduction

Prostate cancer stands as the most prevalent malignancy among males globally and ranks as the fifth leading cause of cancer‐related deaths in men [1]. In Taiwan, there were 7500 newly diagnosed cases of prostate cancer and 1689 deaths attributed to prostate cancer in 2021 [2]. Androgen deprivation therapy (ADT) has been the standard of care for advanced prostate cancer for many years [3]. ADT includes the induction of hypogonadism through orchiectomy, luteinizing hormone‐releasing hormone (LH‐RH) agonist alone or combined with an androgen blockade [4, 5]. Despite its efficacy in prostate cancer treatment and its ability to extend survival, ADT can lead to several troublesome complications, notably an increased risk of osteoporosis or fractures [6, 7]. Cohort studies conducted by the National Health Insurance (NHI) have also validated the heightened fracture risk in patients with prostate cancer undergoing ADT [8, 9]. For patients undergoing long‐term castration therapy for prostate cancer, healthcare providers should remain vigilant about this complication, institute appropriate monitoring, and administer timely osteoporosis medication [9].

The association between single nucleotide polymorphisms (SNPs) and bone events has been extensively studied in the literature [10, 11, 12, 13, 14, 15, 16, 17]. Genome‐wide association studies (GWAS) and meta‐analyses have identified numerous loci associated with bone mineral density (BMD), osteoporosis, and osteoporotic fractures [18, 19, 20, 21]. Recently, the method of Mendelian randomization has gained popularity for identifying causative risk factors for osteoporosis using GWAS results [21, 22].

The concept of polygenic risk score (PRS) has been introduced consequently. PRS is a composite score that incorporates the presence or absence of multiple genetic variants associated with BMD. By weighing the influence of each SNP and summing these SNPs into a PRS, researchers can assess the cumulative contribution of these variants to disease prevalence and incidence in population [11]. For example, the individuals with lower PRS would have lower estimated heel bone mineral density (eBMD) levels, a 17.4‐fold increased risk for osteoporosis and a 1.88‐fold higher risk for bone fracture than the individuals with median score. Thus, genetic predictors could assist in the identification of individuals at risk for bone events, including osteoporosis and fractures [23].

The application of PRS has shown variations among different ethnic groups. The PRS is sensitive to allele frequency and effect size of variants; both can be different between ethnicities. For example, an allele may be common in one ethnicity but may be rare in another ethnicity. Furthermore, population history and inheritance patterns may be different between ethnic groups. Thus, a PRS constructed from one ethnicity may not be applicable to another ethnicity [24]. For instance, a low BMD‐related PGS is associated with up to 2.35‐ and 4.31‐fold increased fracture risk in European and Asian populations [25]. Despite most PRSs in the field of osteoporosis being derived from white populations, there have been studies on ancestry‐specific PRS for the Asian population as well. For instance, a PRS has been shown to be associated with elevated risk of osteoporosis in the Korean population [26]. Furthermore, Kim et al. generated PRS for ten diseases including osteoporosis in East Asia using GWAS data from European and East Asian ancestries and employed six PRS calculation methods [27]. However, there is still no study discussing the association between PRS and risk of bone events for prostate cancer patients receiving ADT, despite the known high risk for osteoporosis in this population.

Herein, we conducted a study using GWAS and data obtained from the Taiwan Precision Medicine Initiative (TPMI) to establish the predictive value of PRS on the risk of bone fracture and osteoporosis among prostate cancer patients receiving ADT.

## 2 Patients and Methods

### 2.1 Study Population

This hospital‐based retrospective cohort study included 57,257 participants aged 20 or older, enrolled in the TPMI project—a nationwide genetic research initiative by Academia Sinica in Taiwan. Medical records and blood samples were collected from Taiwanese volunteers at Taichung Veterans General Hospital between July 2019 and November 2022. The study comprised 903 prostate cancer patients identified by ICD‐9‐CM code 185.x, categorized into ADT and non‐ADT groups. The ADT group included patients who received castration therapy via orchiectomy, LH‐RH agonist, or antagonist. The TCVGH EHR dataset contained comprehensive demographic, clinical, and procedural information. All participants underwent genotyping using the Affymetrix Genome‐Wide TWB 2.0 SNP Array. Written genetic analysis consent was obtained from all participants. The study received approval from the TCVGH Institutional Review Board (IRB No. CE23119A) and adhered to relevant guidelines. Clinical parameters were de‐identified and sourced from electronic medical records.

### 2.2 Genotyping and Quality Control

Blood samples were collected to extract DNA and conduct genotyping using the Axiom Genome‐Wide TWB 2.0 Array Plate (Affymetrix, Santa Clara, CA, USA), containing 714,431 SNPs specifically designed for Taiwan's Han Chinese population [28]. Quality control using Affymetrix Power Tools excluded markers not meeting Hardy–Weinberg equilibrium (*p* < 1.0 × 10^−5^), with minor allele frequency below 0.05, or genotype missing rate > 5%. After quality control, 591,048 SNPs were retained for analysis [29]. Samples with missingness rate > 0.02, inbreeding coefficient > 0.15, or sex mismatch were excluded. Genotype imputation was performed across autosomal chromosomes using the Michigan Imputation Server with the ‘minimac4’ algorithm, utilizing the 1000 Genomes Phase 3 (Version 5) reference panel [30]. Variants with imputation quality INFO score ≥ 0.3 were included in the analysis. Principal component analysis (PCA) was conducted on the imputed genotype data to capture population structure, and the top 10 principal components were included as covariates in downstream analysis. Given the population homogeneity (over 95% Han Chinese ancestry), as previously reported in the TPMI cohort, the risk of substantial population stratification was considered minimal [31].

### 2.3 Polygenic Risk Score Analysis

Polygenic risk scores (PRS) were calculated using PLINK version 2.0, which automatically resolves issues with effect and non‐effect alleles [32]. To prevent multicollinearity, linkage disequilibrium (LD) pruning and clumping were performed to select independent, informative SNPs [33, 34]. LD refers to the non‐random association of alleles at different genetic loci within a population, which can arise due to physical proximity on a chromosome or other evolutionary factors. When two genetic variants, such as SNPs, exhibit strong LD, they are more frequently co‐inherited than would be expected by chance. The PRSs used in this study were derived from the United Kingdom (UK) Biobank, including PGS002315, PGS002387, PGS002436, PGS002534, PGS002583, PGS002632, PGS002681, PGS001274 and PGS001955. The PGS002315, PGS002387, PGS002436, PGS002534, PGS002583, PGS002632, PGS002681 and PGS001955 comprised 1,109,311, 25,831, 53,472, 11,440, 7908, 432,286, 984,276 and 73,565 SNPs, respectively, that were associated with BMD identified solely from individuals of European ancestry and confirmed by a Trans‐ancestry GWAS meta‐analysis [35, 36]. PGS001274 comprised 1270 SNPs, which were associated with osteoporosis identified solely from individuals of European ancestry and confirmed by a Trans‐ancestry GWAS meta‐analysis as well [37]. To calculate a weighted polygenic risk score, the standard equation is as follows:

(1) $${\mathrm{PRS}}_{j}=\sum_{i}^{N}\beta_{i}\times {\text{dosage}}_{\mathrm{ij}}$$

Equation (1) calculates a weighted PRS for individual *j*. *N* represents the total number of SNPs from GWAS used in the PRS calculation. Furthermore, $\beta_{i}$ signifies the effect size of the effect allele of SNP_*i*_, while dosage_*ij*_ is the number of copies of the effect allele in the genotype of individual *j*.

### 2.4 Clinical Parameters and Outcome Definition

Prostate cancer patients were identified using ICD‐9‐CM code 185, confirmed by pathological reports, with the index date defined as the cancer diagnosis date recorded at least twice during outpatient visits or once during hospitalization between January 2009 and January 2022. Comorbidities were extracted from the Taichung Veterans General Hospital (TCVGH) Electronic Health Record (EHR) Database using ICD‐9‐CM and ICD‐10‐CM diagnostic codes for neurologic, pulmonary, connective tissue, chronic kidney, and cardiovascular diseases. Osteoporosis was defined based on medication use, including anti‐osteoporosis drugs, calcium, and vitamin D supplements, identified through Anatomical Therapeutic Chemical (ATC) codes (Table S1).

The study outcome was defined as the occurrence of bone fracture or osteoporosis after the diagnosis of prostate cancer. Fractures and osteoporosis were identified using ICD‐9‐CM and ICD‐10‐CM diagnostic codes documented in the TCVGH EHR database (Table S1). The outcome events included:

- Fractures at common sites such as the femur, vertebrae, and radius.
- Osteoporosis, with or without pathological fracture.

Both inpatient and outpatient diagnoses were included. The complete list of ICD‐9‐CM and ICD‐10‐CM codes used to identify these outcomes is provided in Table S1. All outcome events were required to occur after the initial prostate cancer diagnosis, and data were collected from January 2009 to January 2022.

### 2.5 Statistical Analysis

Hazard ratios (HRs) and 95% confidence intervals (95% CI) were calculated using Cox proportional hazards regression models, with time since study entry as the timescale. Outcomes were censored at loss to follow‐up, death, or end of follow‐up (November 2022). Demographic data were presented as mean ± standard deviation (SD), with continuous variables analyzed by ANOVA and categorical variables by Chi‐square test. Median follow‐up time was analyzed using the Kruskal‐Wallis test. Predictive performance of PRS for bone fracture and osteoporosis was determined by receiver operating characteristic (ROC) curve analysis. Participants were stratified into four quartile groups (Q1–Q4), each representing 25% of the total participants, with Q1 covering 0%–25% PRS values, Q2 covering 26%–50%, Q3 covering 51%–75%, and Q4 covering 76%–100% PRS values. Statistical analyses were performed using SAS version 9.4 and IBM SPSS statistical software version 22.0.

## 3 Results

### 3.1 Baseline Characteristics of the Study Participants

Out of 57,257 TPMI project participants, 903 prostate cancer patients were enrolled, with 457 receiving androgen deprivation therapy (ADT) and 446 not receiving ADT. During follow‐up, 103 fatalities and 62 bone fractures or osteoporosis cases were recorded. The baseline characteristics of both groups are presented in Table 1. The ADT group showed a significantly higher incidence of bone fractures or osteoporosis (8.91% vs. 4.71%, *p* = 0.0113), older age at diagnosis (69.96 vs. 66.81, *p* < 0.0001), higher clinical stage (*p* < 0.0001), and different serum levels of 25(OH)D3, calcium, and phosphorus (*p* = 0.0004, *p* < 0.0001, *p* = 0.0035, respectively). The median follow‐up periods were similar: 5.07 years (IQR 3.21–7.79) for the non‐ADT group and 5.01 years (IQR 2.99–7.47) for the ADT group, with no significant difference between groups.

**TABLE 1:** Characteristics of the patients diagnosed with prostate cancer (*N* = 903).

Variables		Total (*n* = 903)		Without ADT (*n* = 446)		With ADT (*n* = 457)		*p*
		*n*	(%)	*n*	(%)	*n*	(%)	
Age, year	< 65	81	8.97	53	11.88	28	6.13	
	65–75	384	42.52	212	47.53	172	37.64	
	> 75	438	48.5	181	40.58	257	56.24	
Onset age (year)		68.41	8.34	66.81	8.03	69.96	8.34	< 0.0001
BMI	< 18.5	1	0.31	1	0.56	0	0	
	18.5–24	118	36.76	66	36.87	52	36.62	
	≥ 24	202	62.93	112	62.57	90	63.38	
Clinical stage	Stage I	151	16.85	131	29.57	20	4.42	< 0.0001
	Stage II	438	48.88	269	60.72	169	37.31	
	Stage III	116	12.95	30	6.77	86	18.98	
	Stage IV	191	21.32	13	2.93	178	39.29	
PGS002632 (mean/SD)^a^		0.3156	0.4203	0.3323	0.4326	0.2993	0.4078	0.2381
PGS002681 (mean/SD)^a^		−0.2977	0.4374	−0.2697	0.4377	−0.3252	0.4358	0.0565
PGS001955 (mean/SD)^a^		−0.0036	0.086	−0.0007	0.0871	−0.0064	0.0849	0.3246
Comorbidity (*n*/%)^b^	Neurologic disease	66	7.31	31	6.95	35	7.66	0.6828
	Pulmonary disease	61	6.76	28	6.28	33	7.22	0.5724
	Connective tissue disease	5	0.55	4	0.9	1	0.22	0.2121^d^
	Chronic kidney disease	64	7.09	31	6.95	33	7.22	0.8742
	CVD associated disease	255	28.24	139	31.17	116	25.38	0.0536
	Osteoporosis	144	15.95	11	2.47	15	3.28	0.4635
BMD score (mean/SD)^a^								
BMD score	BMD, spine (g/cm^2^)	1.13	0.23	1.17	0.25	1.12	0.23	0.2648
	BMD, left hip (g/cm^2^)	0.81	0.16	0.83	0.13	0.8	0.16	0.2532
	BMD, right hip (g/cm^2^)	0.81	0.14	0.83	0.15	0.8	0.14	0.2687
	T‐score, spine	−0.18	1.98	0.36	2.05	−0.3	1.95	0.0814
	T‐score, left hip	−1.58	1.17	−1.29	1.03	−1.64	1.19	0.1256
	T‐score, right hip	−1.55	1.09	−1.29	1.2	−1.61	1.06	0.1191
Laboratory test	25(OH)D3 (ng/mL)	0.79	4.85	0.21	2.57	1.35	6.28	0.0004
	Calcium (mg/dL)	9.08	0.5	0.09	0.28	0.23	0.42	< 0.0001
	Phosphorus (mg/dL)	3.59	0.77	3.39	0.81	3.66	0.74	0.0035
	Creatinine (mg/dL)	1.12	0.66	1.11	0.45	1.14	0.81	0.4061
Osteoporosis drug (use)	Bisphosphonates	0	0	0	0	0	0	—
	RANKL inhibitor	3	0.33	0	0	3	0.66	0.2493^d^
	SERMs	0	0	0	0	0	0	—
	Parathyroid hormone	0	0	0	0	0	0	—
	Strontium	1	0.11	1	0.22	0	0	0.4939^d^
	Calcium supplement	22	2.44	15	3.36	7	1.53	0.0743
	Vitamin D supplement	0	0	0	0	0	0	—
Outcome (*n*/%)^b^	Bone fracture or osteoporosis	62	6.87	21	4.71	41	8.97	0.0113
Median follow up time (from prostate cancer diagnosis to latest visit) (median/IQR)^c^ (years)		5.05	3.09‐7.74	5.07	3.21–7.79	5.01	2.99–7.67	0.6499

^a^ Continuous variables were expressed as mean ± standard deviation (SD) and were analyzed using ANOVA following a normal data distribution.

^b^ Categorical variables were expressed as numbers (percent) and were analyzed using the Chi‐square test.

^c^ Using Kruskal‐Wallis test.

^d^ Using Fisher's exact test.

### 3.2 Predictive Performance of PRS for the Participants With ADT or Without ADT

**FIGURE 1:**
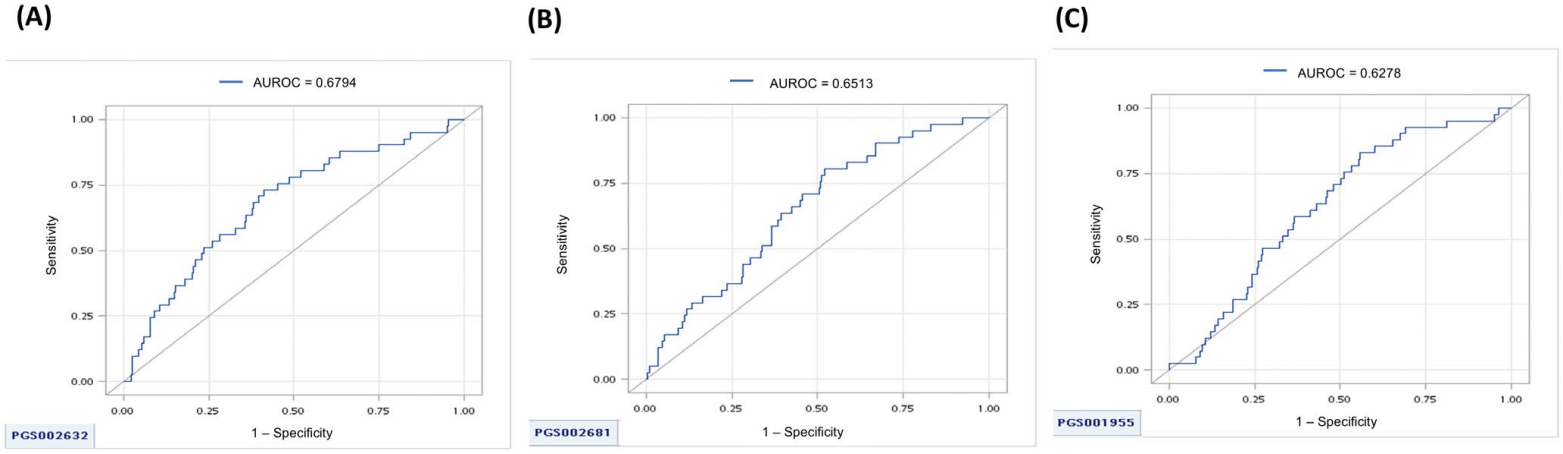
The receiver operating characteristic (ROC) curves for different PRS in predicting bone fracture or osteoporosis in prostate cancer patients receiving androgen deprivation therapy are as follows: (A) The area under the curve (AUC) for PGS002632 is 0.6794, (B) The AUC for PGS002681 is 0.6513, and (C) The AUC for PGS001955 is 0.6278.

The predictive performance of various PRS for bone fracture or osteoporosis was assessed using ROC curve analysis. The AUCs for PRS models, including PGS002315, PGS002387, PGS002436, PGS002534, PGS002583, PGS002632, PGS002681, PGS001274, and PGS001955, ranged from 0.5295 to 0.6794 (Figure 1). Participants were categorized into quartiles (Q1–Q4) based on PGS002632, PGS002681, and PGS001955 scores to evaluate bone fracture or osteoporosis risk, adjusted for clinical covariates (Table 2).

**TABLE 2:** Risk of bone fracture or osteoporosis in the ADT group and without the ADT group.

Variables	ADT (*n* = 457)				Without (*n* = 446)			
	HR	95% CI		*p*	HR	95% CI		*p*
PGS002632								
Q1|Q4	5.934	2.004	17.574	0.0013	2.051	0.542	7.765	0.2903
Q2|Q4	2.85	0.878	9.25	0.0812	1.471	0.393	5.499	0.5666
Q3|Q4	1.844	0.514	6.618	0.3479	2.169	0.619	7.592	0.226
PGS002681								
Q1|Q4	4.863	1.398	16.912	0.0129	3.74	0.967	14.471	0.056
Q2|Q4	4.977	1.404	17.652	0.013	1.751	0.383	8.007	0.4701
Q3|Q4	3.616	0.975	13.405	0.0545	2.12	0.524	8.568	0.2919
PGS001955								
Q1|Q4	4.252	1.21	14.939	0.024	1.409	0.424	4.688	0.5758
Q2|Q4	4.721	1.337	16.672	0.0159	0.907	0.26	3.162	0.878
Q3|Q4	2.796	0.752	10.397	0.125	1.187	0.335	4.208	0.7907

*Note:* Adjusted for age, neurologic disease, pulmonary disease, chronic kidney disease, CVD associated disease, osteoporosis.

Abbreviations: 95% CI, 95% confidence interval; HR, hazard ratio.

**FIGURE 2:**
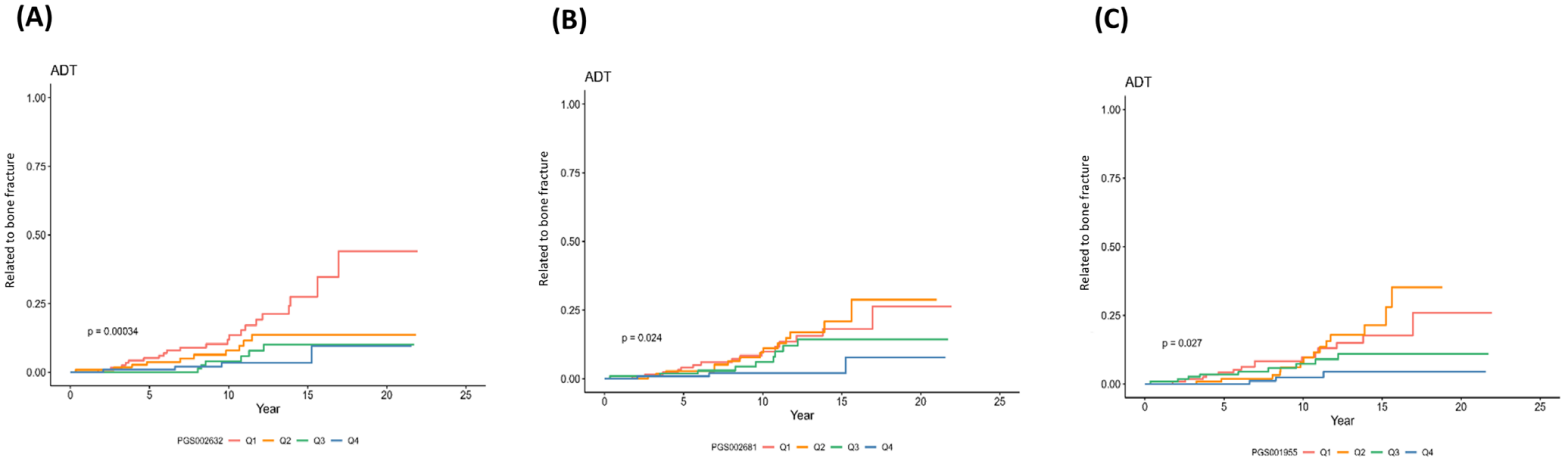
The Kaplan–Meier curve was used to analyze the cumulative incidence of bone fracture or osteoporosis for the 457 prostate cancer patients who received androgen deprivation therapy (ADT) by quartiles of PRS. (A) Cumulative incidence of bone fracture or osteoporosis was statistically significant differences between different quartiles in PGS002632, *p* = 0.00034 (B) Cumulative incidence of bone fracture or osteoporosis was statistically significant differences between different quartiles in PGS002681, *p* = 0.024 (C) Cumulative incidence of bone fracture or osteoporosis was statistically significant differences between different quartiles in PGS001955, *p* = 0.027.

In the ADT group, bone fracture or osteoporosis risk was significantly higher in Q1 compared to Q4 for PGS002632 (HR = 5.934, *p* = 0.0013), PGS002681 (HR = 4.863, *p* = 0.0129), and PGS001955 (HR = 4.252, *p* = 0.024). No significant association was observed in the non‐ADT group. Kaplan–Meier analysis revealed significantly higher cumulative bone fracture or osteoporosis incidence in the lowest PRS quartile for PGS002632 (*p* = 0.00034), PGS002681 (*p* = 0.024), and PGS001955 (*p* = 0.027) in the ADT group (Figure 2). In contrast, no significant differences were found in the non‐ADT group (Figure S2).

### 3.3 Association of PRS and Bone Fracture or Osteoporosis in the ADT Group

Table 3 compares bone fracture or osteoporosis risk between ADT and non‐ADT groups across PRS quartiles, adjusted for clinical covariates. A significant increase in bone fracture or osteoporosis risk was observed in Q1 of PGS002632 (HR = 3.698, *p* = 0.0093) for the ADT group compared to the non‐ADT group. Similar trends were noted in Q1 of PGS002681 and PGS001955 but without statistical significance. In Q2, significant risk increases were found for PGS002681 (HR = 4.611, *p* = 0.0081) and PGS001955 (HR = 4.334, *p* = 0.0071). Kaplan–Meier analysis (Figure S3) showed a significantly higher cumulative bone fracture or osteoporosis incidence in Q1 of PGS002632 for the ADT group (*p* = 0.0032). Figures S4 and S5 revealed similar trends for PGS002681 and PGS001955 in Q2, with significant differences (*p* = 0.016 and *p* = 0.0076, respectively). No significant differences were observed in Q3 and Q4 across all PRS models. In summary, PGS002632 demonstrated the best predictive performance with the highest AUC and HR in identifying bone fracture or osteoporosis risk in the ADT group within the bottom quartile.

**TABLE 3:** Risk of bone fracture or osteoporosis in each quartile of PGS002632, PGS002681 and PGS001955.

Variables	HR	95% CI		*p*
PGS002632				
Q1				
ADT	3.698	1.38	9.912	0.0093
Q2				
ADT	2.414	0.797	7.315	0.1191
Q3				
ADT	1.182	0.366	3.82	0.7796
Q4				
ADT	1.67	0.333	8.362	0.5328
PGS002681				
Q1				
ADT	1.536	0.636	3.709	0.3397
Q2				
ADT	4.611	1.488	14.284	0.0081
Q3				
ADT	2.447	0.813	7.365	0.1115
Q4				
ADT	2.024	0.283	14.452	0.4822
PGS001955				
Q1				
ADT	2.089	0.8	5.458	0.1327
Q2				
ADT	4.334	1.489	12.618	0.0071
Q3				
ADT	2.063	0.678	6.276	0.202
Q4				
ADT	1.141	0.229	5.698	0.8721

*Note:* Adjusted for age, neurologic disease, pulmonary disease, chronic kidney disease, CVD associated disease, osteoporosis.

Abbreviations: 95% CI, 95% confidence interval; HR, hazard ratio.

### 3.4 Subgroup Analysis by Cancer Stage

As bone metastasis could increase the risk of bone fracture and potentially confound the findings, we performed an additional stratified analysis according to cancer stage, dividing patients into stage 1–3 and stage 4 groups. In the stage 1–3 ADT group (*n* = 275), Kaplan–Meier analysis revealed a significantly higher cumulative incidence of bone fracture or osteoporosis among participants in the lowest PRS quartile (Q1) for both PGS002632 (*p* = 0.011) and PGS002681 (*p* = 0.018) (Figure S6). In contrast, no significant differences were observed in the non‐ADT group (*n* = 430). Among patients with stage 4 disease, including the ADT group (*n* = 178) and non‐ADT group (*n* = 13), no significant differences in bone fracture or osteoporosis incidence were found across PRS quartiles (Figure S7). Consequently, our findings indicate that the PRS is capable of predicting the effect of ADT on bone health among prostate cancer patients who do not have bone metastasis.

## 4 Discussion

Our study found that PRS can predict bone fracture or osteoporosis risk in prostate cancer patients receiving ADT in the Han Chinese population using TCVGH‐TPMI cohort data. PGS002632 demonstrated the best predictive performance, with the highest AUC and HR, and a significant bone fracture or osteoporosis risk increase in ADT patients in the lowest quartile (Q1). Patients in the bottom quartile of PGS002632, PGS002681, and PGS001955 had a significantly higher bone fracture or osteoporosis risk when receiving ADT compared to the top quartile, a trend not observed in non‐ADT patients. Additionally, patients in Q1 of PGS002632 and Q2 of PGS002681 and PGS001955 showed increased bone fracture or osteoporosis risk with ADT, highlighting the need for early intervention, such as bone protective agents, in these subgroups. No significant risk difference was observed in higher PRS quartiles (Q3 and Q4).

The PGS Catalog, a novel database and website that compiles PGS data from numerous studies, has been developed to offer researchers a convenient resource for investigating potential associations between diseases and PGSs [38]. For inclusion in the PGS Catalog, any published or preprinted PGS must satisfy two criteria: (1) demonstrate established analytic validity in external samples not used for score development, and (2) provide the necessary information to calculate the score, thereby facilitating a more objective assessment of the PGS's accuracy and enabling external validation of its validity [39].

The PRS PGS002632, comprising 432,286 SNPs, was developed by Weissbrod et al. using European GWAS data from 327,738 individuals in the UK Biobank cohort with the PolyFun‐pred method, which improves cross‐population PRS accuracy despite ancestry differences [36]. PGS002681, derived from the same UK Biobank data, includes 984,276 SNPs using the SBayesR method and focuses on bone mineral density [36]. PGS001955, based on data from 391,124 individuals of nine ancestries in the UK Biobank, analyzed 73,565 variants and demonstrated effectiveness in predicting bone mineral density and bone fracture or osteoporosis risk [35]. Additionally, we discovered that it could also correlate with the risk of bone fracture or osteoporosis in prostate cancer patients undergoing hormone therapy.

Osteoporosis and bone fractures are common among the elderly, leading to increased morbidity and mortality. Identifying individuals at high risk for fractures is crucial for early intervention. Androgen deprivation therapy (ADT) for prostate cancer, while improving survival, significantly increases the risk of bone fracture or osteoporosis, including osteoporosis and fractures. Studies show ADT accelerates bone loss and raises fracture risk by 1.5‐fold, with fracture‐related hospitalizations increasing by 1.7‐fold [6, 7]. A landmark 2005 study reported fractures in 19.4% of patients on ADT versus 12.6% of those not receiving ADT (*p* < 0.001) [7]. Recent studies in Asia corroborate these findings. A Korean nationwide cohort study linked ADT to increased osteoporosis and fracture risk in prostate cancer patients [40]. Similarly, Wu et al. demonstrated a 14.4% fracture risk in Taiwanese patients on ADT compared to 7.1% without ADT [8]. Additional research using Taiwan's National Health Insurance Research Database found that surgical castration posed the highest bone fracture or osteoporosis risk, followed by medical castration, while osteoporosis medication significantly reduced fracture risk [9]. Androgen deprivation therapy for prostate cancer conclusively increases the risk of bone mass loss, bone fractures, and osteoporosis, which can lead to a deterioration in body function and quality of life. This, in turn, may result in a decline in performance status and an increased risk of mortality.

Osteoporosis, a widespread skeletal disease, is influenced by factors such as diet, activity, endocrine status, comorbidities, and genetics. GWAS studies have identified numerous genetic loci linked to BMD, osteoporosis, and fractures [21]. While single‐gene analyses offer limited insights, polygenic risk scores (PRS) integrate multiple genetic variants, enhancing predictions of BMD and bone fracture or osteoporosis risk.

Most osteoporosis PRSs are derived from European populations. Xiao et al. examined four BMD‐related PRSs in the UK Biobank cohort, finding the best PRS predicted fracture risk with HRs of 1.24 (European ancestry), 1.28 (African ancestry), and 1.34 (Asian ancestry), showing effectiveness for Asians but not African [25]. Kim et al. demonstrated PRSs from European and East Asian GWAS data effectively predicted osteoporosis risk in Koreans [27]. A study from our institute using the TPMI‐VGHTC cohort found lower PRS correlated with lower BMD, higher fracture rates, and elevated FRAX scores, supporting PRS as a valuable tool to refine fracture risk prediction, especially for moderate to low‐risk individuals [41].

Although PRS are widely studied for prostate cancer susceptibility, no studies have focused on bone fracture or osteoporosis risk, which increases during hormone therapy for prostate cancer [42, 43]. Hook et al. found that PRS could predict fracture risk in breast cancer patients on aromatase inhibitors, independent of BMD [44]. Our study is the first to associate PRS with bone fracture or osteoporosis risk in prostate cancer patients undergoing ADT, highlighting the potential of PRS for personalized management strategies.

To further clarify whether the observed bone fracture or osteoporosis was primarily related to bone fragility or bone metastases, we conducted a subgroup analysis stratified by prostate cancer stage. In the stage 1–3 ADT group (non‐metastatic disease), participants in the lowest PRS quartile (Q1) for both PGS002632 and PGS002681 exhibited significantly higher cumulative bone fracture or osteoporosis incidence compared with other quartiles. This finding suggests that PRS effectively captured genetic susceptibility to fragility‐related skeletal events in patients receiving ADT. In contrast, no significant associations were observed in the stage 1–3 non‐ADT group, supporting the role of ADT exposure as an environmental trigger that interacts with genetic predisposition. Among patients with stage 4 disease, no significant differences were detected across PRS quartiles, indicating that bone fracture or osteoporosis in advanced prostate cancer is likely driven by bone metastasis rather than underlying skeletal fragility. However, the interpretation of results in stage 4 disease may be limited because almost all patients in this stage currently receive ADT, making subgroup comparisons difficult. Together, these results strengthen the interpretation that PRS mainly predicts osteoporosis‐ or ADT‐related bone fracture or osteoporosis risk rather than metastasis‐related events.

This study has several limitations. First, bone fractures and osteoporosis were identified solely through ICD‐9‐CM and ICD‐10‐CM diagnostic codes documented in the TCVGH EHR database. Traditional definitions of skeletal‐related complications in clinical trials also include radiation to bone, surgery to bone, and spinal cord compression; however, these procedures are not consistently or specifically coded in administrative databases and therefore could not be captured in this study. Regarding osteoporosis, although BMD and T‐score measurements were available for a subset of patients, these assessments were limited to those with clinical indications, introducing potential selection bias. To capture a broader clinically recognized population, osteoporosis was defined using diagnostic codes and osteoporosis medication use. This approach may not identify undiagnosed or untreated subclinical cases, potentially reducing diagnostic precision. Nevertheless, it captures clinically recognized bone fracture and osteoporosis events relevant for assessing skeletal complications. As a result, our findings primarily reflect clinically documented bone fractures and osteoporosis rather than the full spectrum of skeletal complications observed in clinical practice.

Second, although we conducted a subgroup analysis stratified by prostate cancer stage to differentiate metastatic from non‐metastatic cases, residual misclassification of bone fracture events remains possible. Because these outcomes were identified solely through administrative diagnostic codes, we could not fully distinguish fractures resulting from bone metastases from those due to osteoporosis or androgen deprivation therapy (ADT). Additionally, staging and coding information may be incomplete or delayed in real‐world databases, potentially leading to partial overlap between metastatic and non‐metastatic cases. In addition, administrative staging and coding may be incomplete or delayed, potentially leading to misclassification of some metastatic cases. Furthermore, information on the use of bone‐protective agents such as bisphosphonates or denosumab was incomplete in our dataset. Therefore, we were unable to adjust for these medications, which may influence the risk of bone fractures or osteoporosis. These limitations should be considered when interpreting the association between PRS and skeletal fragility risk. Third, the relatively small sample size and short follow‐up period may underestimate the long‐term risk of bone fractures or osteoporosis and limit the generalizability of the findings regarding disease treatment and prognosis.

The participants were patients from our institute, and the PRS used was derived from European ancestry GWAS, potentially reducing its predictive accuracy for the Han Chinese population. To improve the transferability of genetic findings, future studies should increase diversity during the discovery phase of PRS development. Additionally, the study did not analyze different types of ADT (e.g., surgical castration, GnRH agonists/antagonists) due to the limited sample size.

Most importantly, although bone metastasis may be associated with an increased risk of fractures, we did not exclude patients with bone metastasis to avoid overestimating ADT‐related osteoporosis. Our analysis revealed a higher proportion of patients at stages 2 and 4, suggesting that clinical staging correction may not be necessary. This observation implies that the impact of stage IV disease prevalence on our study's assessment of ADT‐related osteoporosis may be relatively minor. Finally, bone protective agents are recommended for the prevention of skeleton‐related events in patients with metastatic castration‐resistant prostate cancer [45, 46, 47, 48, 49]. Bisphosphates showed a significant effect in preventing fractures (risk ratio [RR], 0.80; *p* = 0.005) and osteoporosis (RR, 0.39; *p* < 0.00001) [45]. However, since bone protective agents are not routinely used for prostate cancer patients treated with ADT, it is challenging to assess their impact accurately.

In clinical practice, integrating PRS with conventional clinical risk factors may help identify prostate cancer patients at higher risk of treatment‐related bone loss, allowing earlier bone mineral density (BMD) screening and timely preventive interventions, such as calcium/vitamin D supplementation or bisphosphonate therapy. As a future direction, developing a GWAS‐based PRS model specifically designed for the Taiwanese population can be achieved by utilizing national‐level data from the TPMI study cohort, conducted by Academia Sinica in collaboration with 13 medical center‐level hospitals in Taiwan. Utilizing this comprehensive dataset will improve the precision of predicting disease incidence and prognosis for the Taiwanese population.

## 5 Conclusion

This hospital‐based cohort study observed that PRS could predict the risk of bone fracture or osteoporosis for prostate cancer patients receiving ADT among Han Chinese individuals. It could serve as a biomarker for prostate cancer patients starting ADT and be a guide for managing bone fracture or osteoporosis and initiating early bone protective agents.

## Author Contributions

Study design and protocol development: Y.‐Q.L., J.‐R.L., S.‐C.H., I.‐C.C.; manuscript writing and editing: Y.‐Q.L., L.‐W.C., S.‐C.H., I.‐C.C.; statistical analysis: I.‐C.C., H.‐W.Y.; data collection and patient management: S.‐C.H., L.‐W.C.; supervision or mentorship: S.‐C.H. and I.‐C.C. All authors reviewed the final manuscript.

## Ethics Statement

The studies involving human participants were reviewed and approved by certification at Taichung Veteran General Hospital, Taiwan, with certification of approval with IRB: CE23119A.

## Consent

The patients provided their written informed consent to participate in this study.

## Conflicts of Interest

The authors declare no conflicts of interest.

## Supporting information


**FIGURE S1:** Flow chart for enrolled participants in the study.

**FIGURE S2:** The Kaplan–Meier curve was used to analyze the cumulative incidence of bone fracture or osteoporosis for the 446 prostate cancer patients who did not receive androgen deprivation therapy (ADT) by quartiles of PRS. (A) cumulative incidence of bone fracture or osteoporosis was no statistically significant differences between different quartiles in PGS002632, *p* = 0.57 (B) cumulative incidence of bone fracture or osteoporosis was no statistically significant differences between different quartiles in PGS002681, *p* = 0.22 (C) cumulative incidence of bone fracture or osteoporosis was no statistically significant differences between different quartiles in PGS001955, *p* = 0.89.

**FIGURE S3:** The Kaplan–Meier curve was used to analyze the cumulative incidence of bone fracture or osteoporosis in prostate cancer patients with or without androgen deprivation therapy (ADT) across each quartile of PGS002632. The results were as follows: (A) For prostate cancer patients in Q1 of PGS002632, the risk of bone fracture or osteoporosis was significantly higher for those with ADT compared to those without ADT (*p* = 0.0032). (B) For patients in Q2 of PGS002632, the risk of bone fracture or osteoporosis did not differ between those with and without ADT (*p* = 0.14). (C) For patients in Q3 of PGS002632, the risk of bone fracture or osteoporosis was also not different between the two groups (*p* = 0.77). (D) Similarly, for patients in Q4 of PGS002632, there was no difference in the risk of bone fracture or osteoporosis between those with and without ADT (*p* = 0.84).

**FIGURE S4:** The Kaplan–Meier curve was used to analyze the cumulative incidence of bone fracture or osteoporosis in prostate cancer patients with or without androgen deprivation therapy (ADT) across each quartile of PGS002681. The results were as follows: (A) For prostate cancer patients in Q1 of PGS002681, the risk of bone fracture or osteoporosis did not differ between those with and without ADT (*p* = 0.32). (B) For patients in Q2 of PGS002681, the risk of bone fracture or osteoporosis was significantly higher for those with ADT compared to those without ADT (*p* = 0.016). (C) For patients in Q3 of PGS002681, the risk of bone fracture or osteoporosis was also not different between the two groups (*p* = 0.21). (D) Similarly, for patients in Q4 of PGS002681, there was no difference in the risk of bone fracture or osteoporosis between those with and without ADT (*p* = 0.95).

**FIGURE S5:** The Kaplan–Meier curve was used to analyze the cumulative incidence of bone fracture or osteoporosis in prostate cancer patients with or without androgen deprivation therapy (ADT) across each quartile of PGS001955. The results were as follows: (A) For prostate cancer patients in Q1 of PGS001955, the risk of bone fracture or osteoporosis did not differ between those with and without ADT (*p* = 0.096). (B) For patients in Q2 of PGS001955, the risk of bone fracture or osteoporosis was significantly higher for those with ADT compared to those without ADT (*p* = 0.0076). (C) For patients in Q3 of PGS001955, the risk of bone fracture or osteoporosis was also not different between the two groups (*p* = 0.28). (D) Similarly, for patients in Q4 of PGS001955, there was no difference in the risk of bone fracture or osteoporosis between those with and without ADT (*p* = 0.56).

**FIGURE S6:** Kaplan–Meier curves for cumulative incidence of bone fracture or osteoporosis in Stage 1–3 (non‐metastatic) prostate cancer patients stratified by PRS quartiles. Analyses were performed separately for patients receiving ADT and those not receiving ADT.

**FIGURE S7:** Kaplan–Meier curves for cumulative incidence of bone fracture or osteoporosis in Stage 4 (metastatic) prostate cancer patients stratified by PRS quartiles, separately for patients receiving ADT and non‐ADT patients. No significant differences were observed among PRS quartiles in either group.

**TABLE S1:** Clinical parameters and outcome evaluation.

## Data Availability

All data used in this study are available in this article. However, the individual‐level PRS and prostate cancer information data are not currently available within the paper. However, we are committed to facilitating access to the data for interested researchers. To request access to the underlying data, please contact the corresponding author. We will provide further information regarding the availability and any necessary procedures for obtaining access, taking into account any ethical, legal, or privacy considerations associated with the data. We appreciate your understanding and patience in this matter.
