# Supplementary figures and images for: Polygenic Risk Score as a Predictor of Bone Fracture or Osteoporosis in Prostate Cancer Patients Receiving Androgen Deprivation Therapy
Source: Cancer Med. 2025 Nov 20;14(22):e71395. doi: 10.1002/cam4.71395 (PMC12631746; doi:10.1002/cam4.71395)

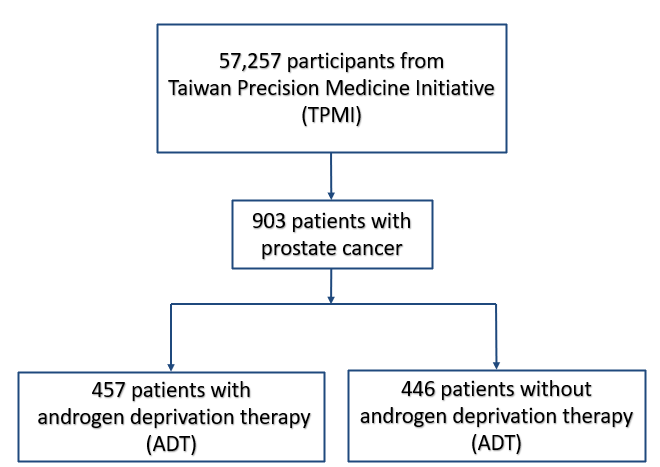

Supplement: Supplementary file 1 — FIGURE S1: Flow chart for enrolled participants in the study. [file CAM4-14-e71395-s001.tif]

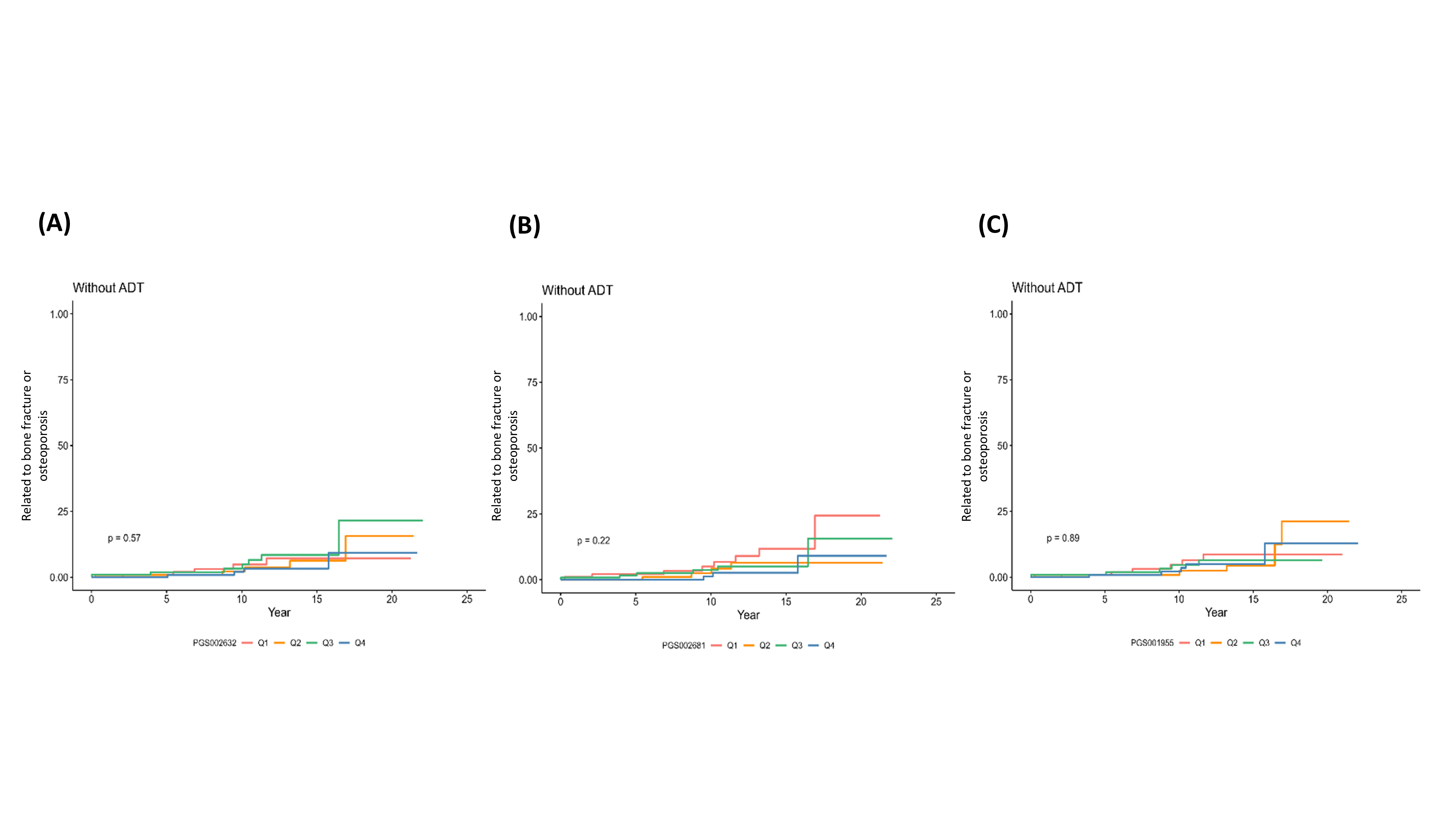

Supplement: Supplementary file 2 — FIGURE S2: The Kaplan–Meier curve was used to analyze the cumulative incidence of bone fracture or osteoporosis for the 446 prostate cancer patients who did not receive androgen deprivation therapy (ADT) by quartiles of PRS. (A) cumulative incidence of bone fracture or osteoporosis was no statistically significant differences between different quartiles in PGS002632, p = 0.57 (B) cumulative incidence of bone fracture or osteoporosis was no statistically significant differences between different quartiles in PGS002681, p = 0.22 (C) cumulative incidence of bone fracture or osteoporosis was no statistically significant differences between different quartiles in PGS001955, p = 0.89. [file CAM4-14-e71395-s003.tif]

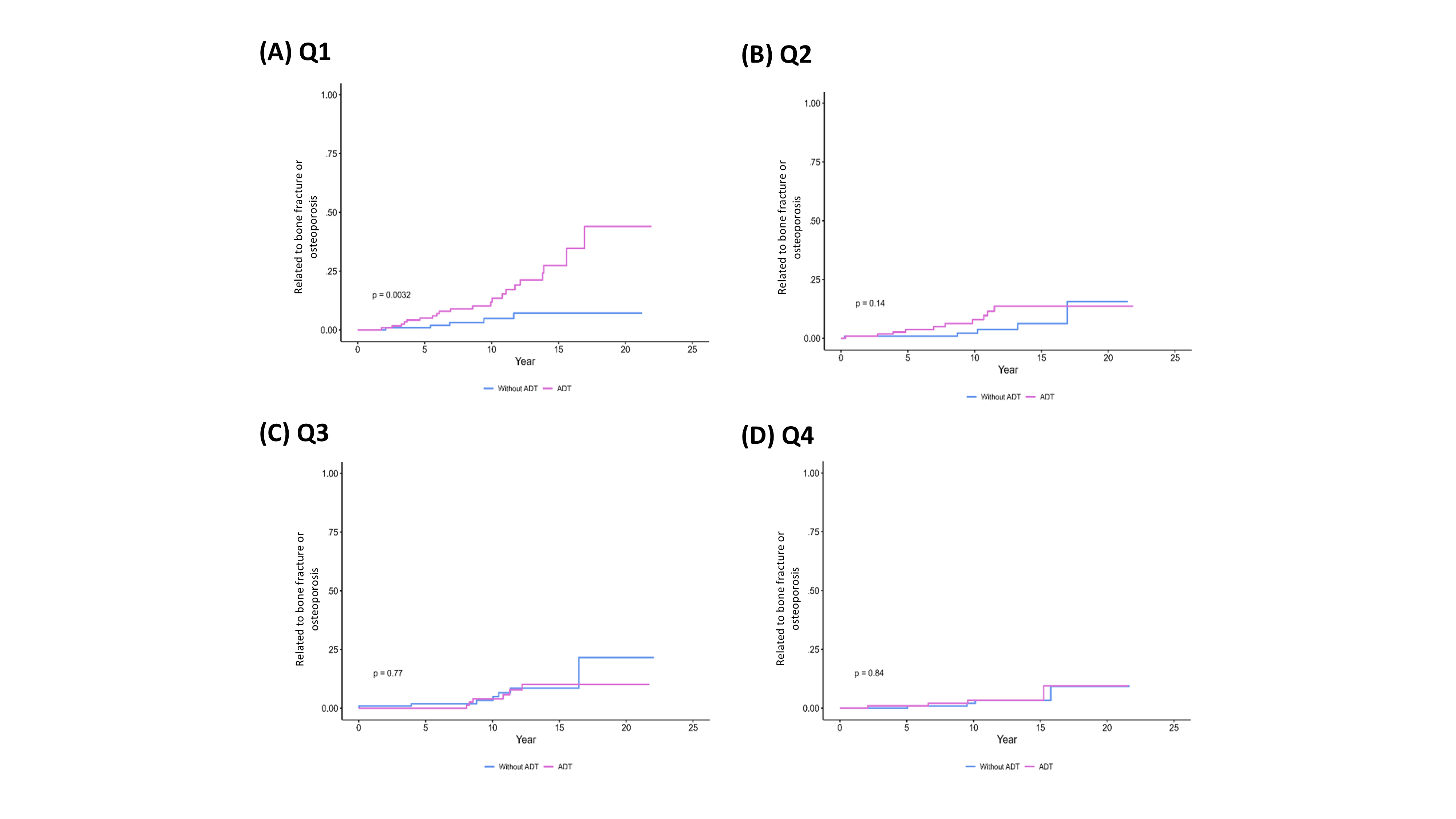

Supplement: Supplementary file 3 — FIGURE S3: The Kaplan–Meier curve was used to analyze the cumulative incidence of bone fracture or osteoporosis in prostate cancer patients with or without androgen deprivation therapy (ADT) across each quartile of PGS002632. The results were as follows: (A) For prostate cancer patients in Q1 of PGS002632, the risk of bone fracture or osteoporosis was significantly higher for those with ADT compared to those without ADT (p = 0.0032). (B) For patients in Q2 of PGS002632, the risk of bone fracture or osteoporosis did not differ between those with and without ADT (p = 0.14). (C) For patients in Q3 of PGS002632, the risk of bone fracture or osteoporosis was also not different between the two groups (p = 0.77). (D) Similarly, for patients in Q4 of PGS002632, there was no difference in the risk of bone fracture or osteoporosis between those with and without ADT (p = 0.84). [file CAM4-14-e71395-s006.tif]

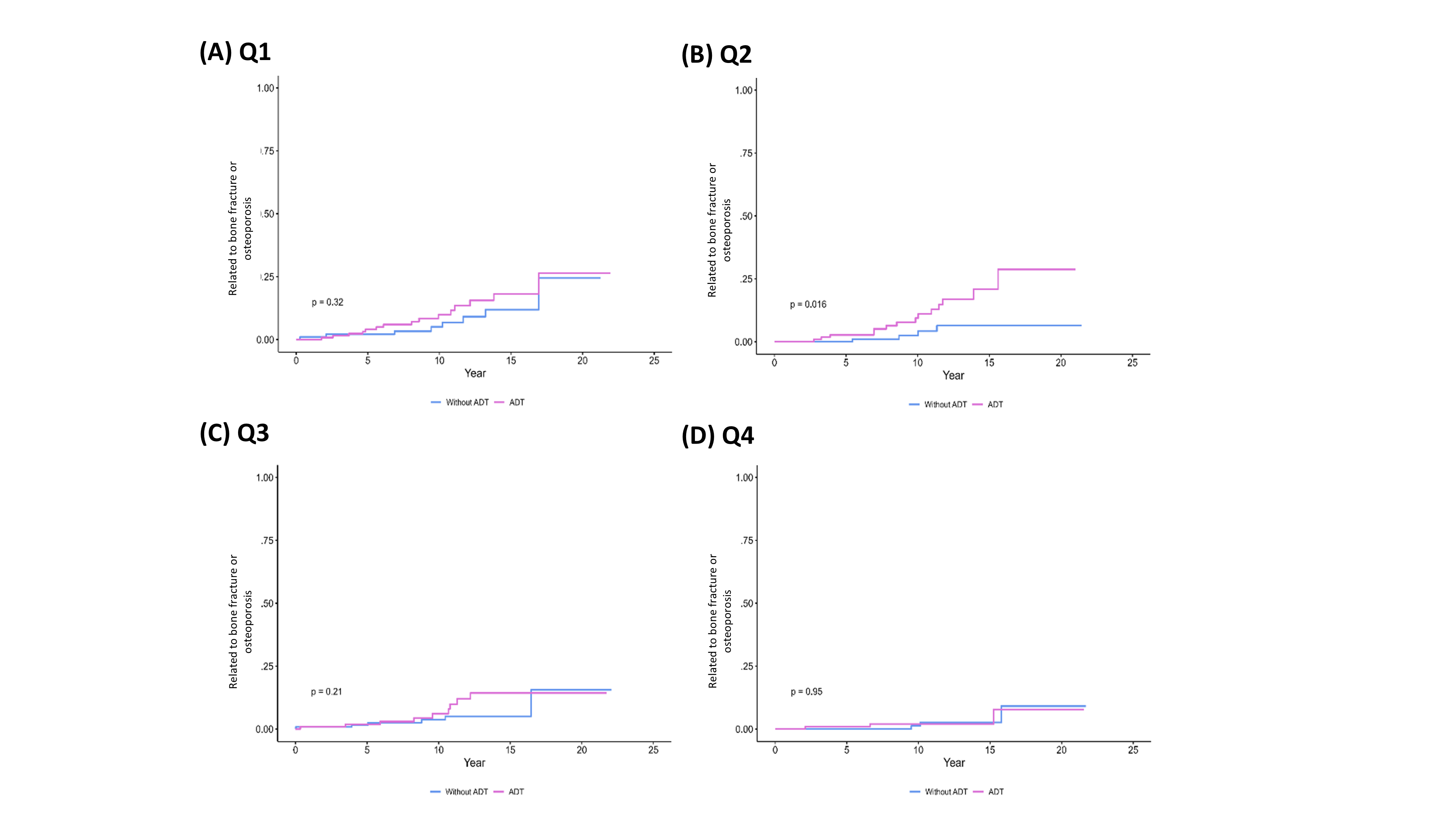

Supplement: Supplementary file 4 — FIGURE S4: The Kaplan–Meier curve was used to analyze the cumulative incidence of bone fracture or osteoporosis in prostate cancer patients with or without androgen deprivation therapy (ADT) across each quartile of PGS002681. The results were as follows: (A) For prostate cancer patients in Q1 of PGS002681, the risk of bone fracture or osteoporosis did not differ between those with and without ADT (p = 0.32). (B) For patients in Q2 of PGS002681, the risk of bone fracture or osteoporosis was significantly higher for those with ADT compared to those without ADT (p = 0.016). (C) For patients in Q3 of PGS002681, the risk of bone fracture or osteoporosis was also not different between the two groups (p = 0.21). (D) Similarly, for patients in Q4 of PGS002681, there was no difference in the risk of bone fracture or osteoporosis between those with and without ADT (p = 0.95). [file CAM4-14-e71395-s007.tif]

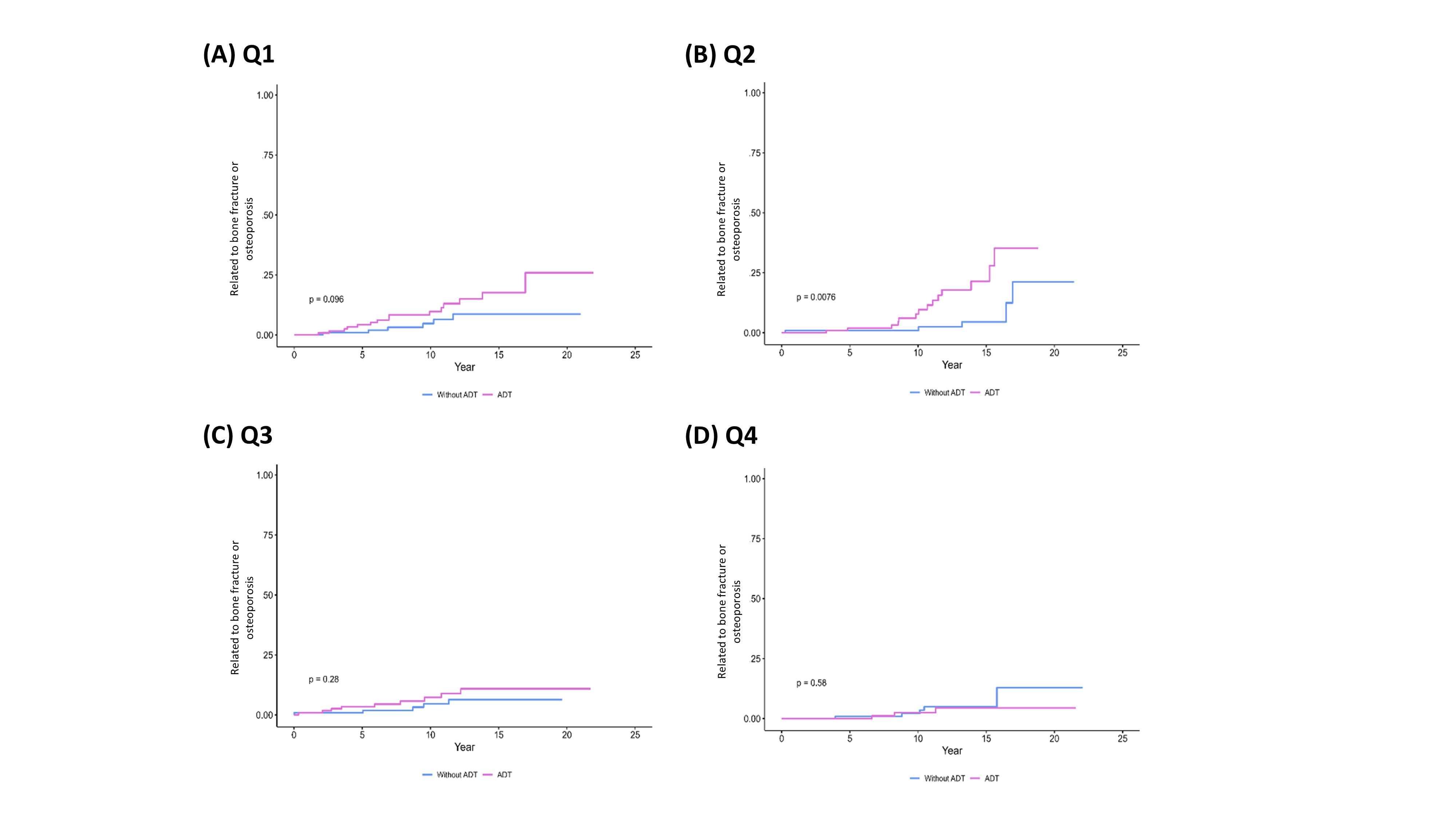

Supplement: Supplementary file 5 — FIGURE S5: The Kaplan–Meier curve was used to analyze the cumulative incidence of bone fracture or osteoporosis in prostate cancer patients with or without androgen deprivation therapy (ADT) across each quartile of PGS001955. The results were as follows: (A) For prostate cancer patients in Q1 of PGS001955, the risk of bone fracture or osteoporosis did not differ between those with and without ADT (p = 0.096). (B) For patients in Q2 of PGS001955, the risk of bone fracture or osteoporosis was significantly higher for those with ADT compared to those without ADT (p = 0.0076). (C) For patients in Q3 of PGS001955, the risk of bone fracture or osteoporosis was also not different between the two groups (p = 0.28). (D) Similarly, for patients in Q4 of PGS001955, there was no difference in the risk of bone fracture or osteoporosis between those with and without ADT (p = 0.56). [file CAM4-14-e71395-s005.tif]

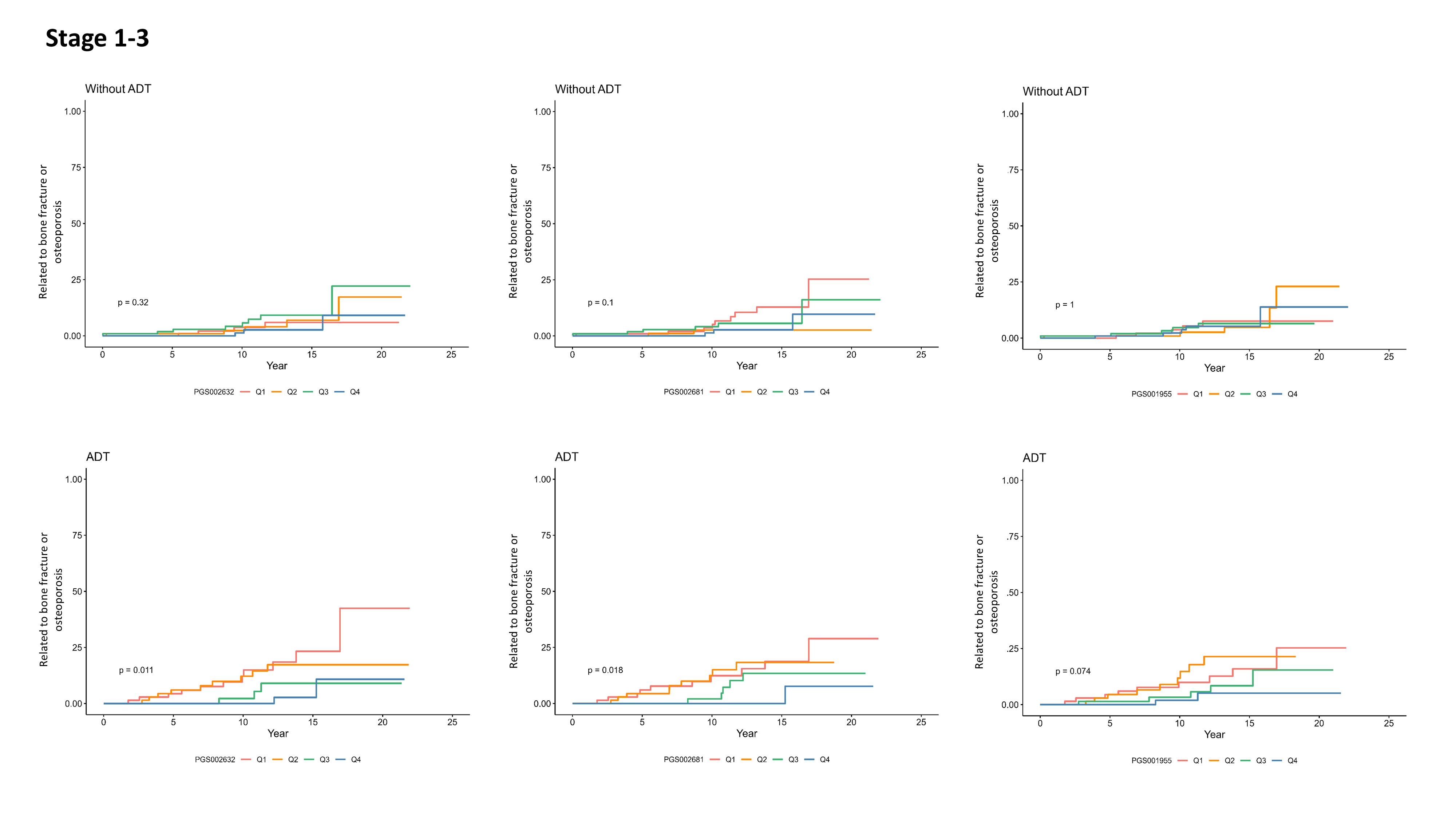

Supplement: Supplementary file 6 — FIGURE S6: Kaplan–Meier curves for cumulative incidence of bone fracture or osteoporosis in Stage 1–3 (non‐metastatic) prostate cancer patients stratified by PRS quartiles. Analyses were performed separately for patients receiving ADT and those not receiving ADT. [file CAM4-14-e71395-s008.tif]

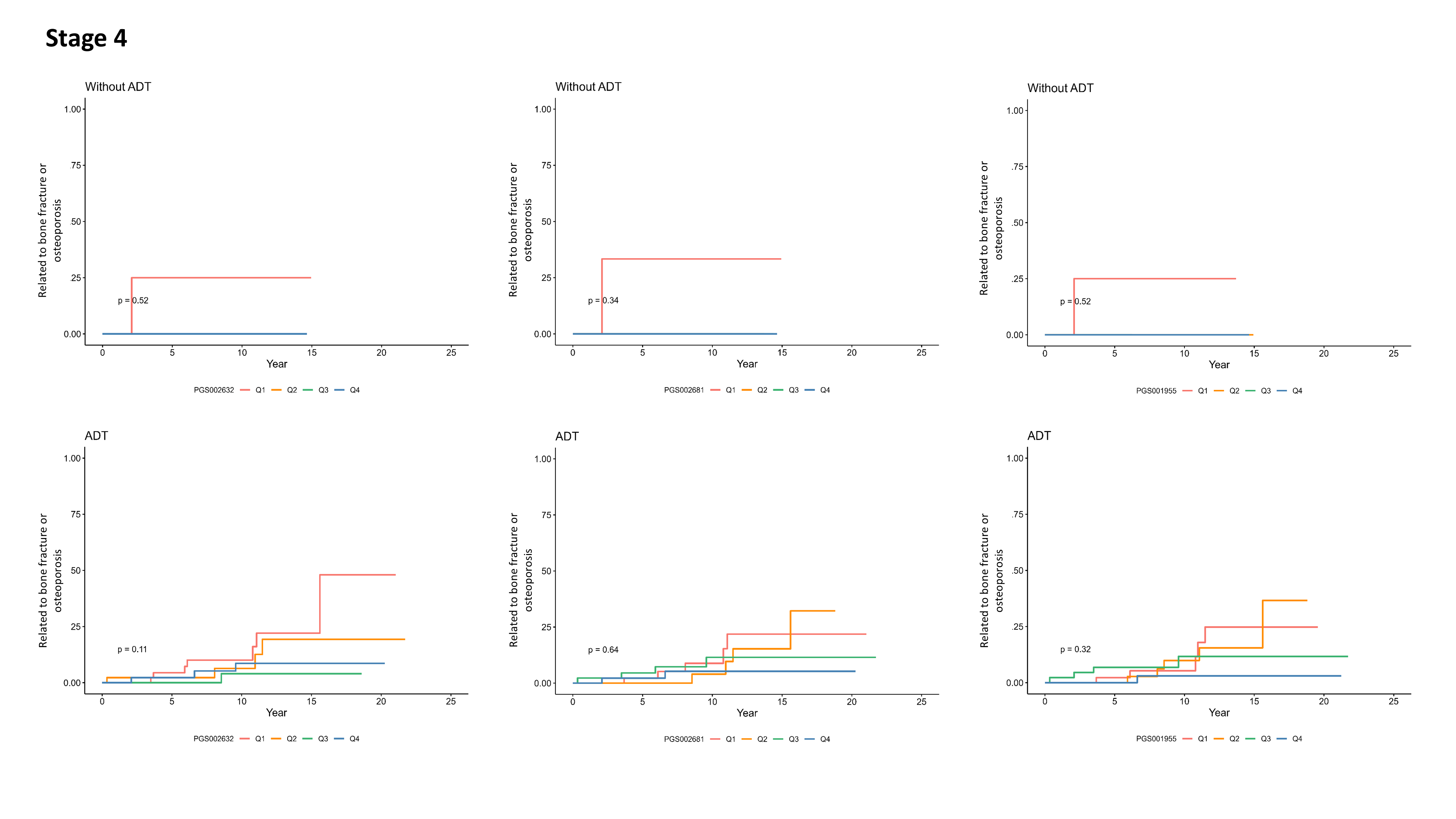

Supplement: Supplementary file 7 — FIGURE S7: Kaplan–Meier curves for cumulative incidence of bone fracture or osteoporosis in Stage 4 (metastatic) prostate cancer patients stratified by PRS quartiles, separately for patients receiving ADT and non‐ADT patients. No significant differences were observed among PRS quartiles in either group. [file CAM4-14-e71395-s002.tif]
