# Supplementary material for: Polygenic Risk Score as a Predictor of Bone Fracture or Osteoporosis in Prostate Cancer Patients Receiving Androgen Deprivation Therapy
Source: Cancer Med. 2025 Nov 20;14(22):e71395. doi: 10.1002/cam4.71395 (PMC12631746; doi:10.1002/cam4.71395)
Supplement: Supplementary file 8 — TABLE S1: Clinical parameters and outcome evaluation. [file CAM4-14-e71395-s004.docx]

**Supplementary Table 1.** Clinical parameters and outcome evaluation.

| Category | Details |
| --- | --- |
| Study Population | Prostate cancer patients identified based on ICD-9-CM code 185 and confirmed by pathological reports. |
| Index Date | Defined as the date of prostate cancer diagnosis, determined using ICD-9-CM code 185 recorded at least twice in outpatient visits or once during hospitalization between January 2009 and January 2022. |
| Data Source | Pertinent biochemical data were extracted from the TCVGH database. |
| Covariates | Age, body mass index (BMI), and comorbidities. |
| Comorbidity Data | Obtained from electronic health records of TCVGH using ICD-9 diagnostic codes. |
| Comorbidity Categories | - **Neurologic diseases**: Stroke, Parkinson’s disease, paraplegia, and dementia (ICD-9-CM: 430-438, 332.0, 332.1, 342, 3441, 290, 331.0, 331.2, A210) - **Pulmonary diseases**: ICD-9-CM: 490-496, 500-505 - **Connective tissue diseases**: ICD-9-CM: 7100, 7101, 7104, 7140-7142, 71481, 5171, 725 - **Chronic kidney diseases**: ICD-9-CM: 403, 404, 582, 583, 585, 586, 588, V42.0, V45.1, V56, A350 - **Cardiovascular diseases** (excluding stroke): ICD-9-CM: 427.31, 428, 433.10, 433.11, 441, 4439, 7854, V434, 413.9, 410, 412, 414.00-414.05, 414.8, 401.0, 401.2, 401.9, 402.00, 402.01, 402.10, 402.11, 402.90, 402.91, 250, 268.9, 272.0, 272.1, 272.4, 277.7, 790.21, 790.29 |
| Definition of Osteoporosis | Based on the use of osteoporosis-related medications, including anti-osteoporosis drugs, calcium supplements, and vitamin D supplements, obtained using Anatomical Therapeutic Chemical (ATC) codes from the TCVGH electronic health records. |
| Primary Analysis | - Incidence of skeletal-related events (SRE) among prostate cancer participants. - Correlation between genetic profiles and clinical parameters, including age at diagnosis, BMI, clinical stage, BMD score, and medical history. |
| Outcome Evaluation | - Bone fracture or osteoporosis after prostate cancer diagnosis confirmed using ICD-9-CM codes, including osteoporosis (733.00) and fractures (813.4, 820.8, 820.00, 820.02, 820.03, 820.20, 820.22, 805.2, 805.4, 733.0+733.14, 733.0+733.13, 733.09+733.14, 733.09+733.13). - Follow-up period: January 2009 to January 2022. |
